# Examining secondary school students’ views of model evaluation through an integrated framework of personal epistemology

**DOI:** 10.1007/s11251-021-09534-9

**Published:** 2021-02-24

**Authors:** Silvia Wen-Yu Lee, Hsin-Kai Wu, Hsin-Yi Chang

**Affiliations:** 1 Graduate Institute of Information and Computer Education, National Taiwan Normal University, No. 162, Sec. 1, Heping E. Rd., Taipei, 106 Taiwan, ROC; 2 Graduate Institute of Science Education, National Taiwan Normal University, No. 162, Sec. 1, Heping E. Rd., Taipei, 106 Taiwan, ROC; 3 Program of Learning Sciences, Institute for Research Excellence in Learning Sciences, National Taiwan Normal University, No. 162, Sec. 1, Heping E. Rd., Taipei, 106 Taiwan, ROC

**Keywords:** Model evaluation, Personal epistemology, Scientific model, Secondary school students

## Abstract

The aim of the study was to investigate students’ views of model evaluation through the lens of personal epistemology. We developed an integrated analytical framework by combining a developmental framework, including absolutist, multiplist, and evaluatist, with a multi-dimensional framework, including limits of knowing, certainty of knowing, and criteria of knowing. Furthermore, we examined the potential influence of the question contexts and the students’ grade levels. A total of 188 secondary school students were surveyed. Students answered two sets of model evaluation questions based on two scientific contexts. After reading the information about the two models, the students had to choose from three epistemic assumptions and then provide written justifications explaining their choice of assumptions. Quantitative and qualitative analyses were conducted for the multiple-choice questions and the written responses. In both contexts there were higher percentages of 11th-grade students choosing the evaluatist assumptions than the eighth-grade students. For students choosing multiplist and evaluatist assumptions, the 11th-grade students were more likely than the eighth-grade students to think in terms of pragmatic and evidential criteria as *the criteria of knowing*. Different contexts of the questions evoked different views of model evaluation particularly regarding the *limits of knowing*. Four additional categories of epistemic levels also emerged from the data. This study provides a new framework for understanding students’ thinking about model evaluation. Implications and suggestions for future research are provided.

## Introduction

Students’ learning of scientific models and modeling is one of the major goals of science teaching (National Research Council 2007, 2012). Teaching scientific models and modeling entails not only teaching of science content, but more importantly, providing opportunities for students to learn modeling practices and to develop views of scientific models and modeling (Gobert et al. 2011; Prins et al. 2010). Research generally relates students’ views of scientific models and modeling to the understanding of the nature, purpose, and process of modeling (Grosslight et al. 1991; Schwarz and White 2005). While modeling-based activities can help students enrich and refine their epistemological understanding of models and modeling (Tasquier et al. 2016), students’ views of models and modeling also play an important role in their learning. Researchers have found that students’ advanced views of models and modeling have an impact on their affective dimension of learning such as their engagement in modeling activities (Gobert and Discenna 1997; Gobert et al. 2011). Students’ views of models and modeling also influence their learning of science content (Soulios and Psillos 2016; Treagust et al. 2002) and other science performance such as scientific explanations (Baumfalk et al. 2018) and modeling practices (Sins et al. 2009).

Model evaluation is one of the important aspects of scientific modeling, which is described as the iterative processes of model construction, model evaluation, model revision, and model use (National Research Council 2012). Model evaluation concerns the questions: “Is there a way to decide whether one model is better than another?” and “What are the criteria for the evaluation of a model?” (Schwarz and White 2005; Sins et al. 2009). It is informed by scientists’ practices that model evaluation should be based on scientific evidence and the purposes of modeling, and models are validated by comparing models to observations and measurements in the real world (Grosslight et al. 1991; Schwarz et al. 2009). Other concepts which relate to model evaluation include model testing (Grünkorn et al. 2014) and model validation (Crawford and Cullin 2005). Although different research instruments have been created for measuring students’ views of models and modeling, not all of these instruments include model evaluation as a sub-construct. Compared to other more commonly researched aspects such as the nature and purpose of models and the change of models (Soulios and Psillos 2016), students’ views of model evaluation have been overlooked in the science educational research.

Most researchers agree that students’ epistemic views of models and modeling are a subset of the epistemic beliefs of science (Soulios and Psillos 2016). However, in most studies, the use of the term epistemology was not operationalized in the coding categories for views of models and modeling. Only a few researchers have described the lower levels of understanding of models and modeling as naïve realism and the higher levels as sophisticated (Soulios and Psillos 2016; Tasquier et al. 2016). So far, few studies have conducted in-depth analyses of students’ understanding of model evaluation by using a comprehensive framework of personal epistemology. Personal epistemology is defined as “what individuals think knowledge is and how they think that they and others know” (p. 227). Different personal epistemological frameworks such as Hofer’s (2000) four dimensions of epistemic beliefs and Kuhn’s (1993) three epistemic positions have been adopted in science educational research for investigating students’ beliefs about science (Lee et al. 2016; Lee and Tsai 2012) and how students learn science (Kuhn 1993; Yang and Tsai 2010). Yet, these frameworks have rarely been used in studies of scientific modeling.

Therefore, in this study, we developed an integrated framework of personal epistemology by combining the categories developed by Kuhn and colleagues (Kuhn 1999; Kuhn and Park 2005; Kitchener 1983). Kuhn’s stage-based framework provides three distinct levels of epistemic development. Nevertheless, it is only along the one dimension of epistemic views, from an objective to a subjective view of knowledge. In order to provide a broader sense of progression, a multidimensional framework is also needed. By drawing on the dimensional and developmental frameworks, an integrated view of personal epistemology allows further analysis of the students’ or teachers’ epistemic patterns (Feucht 2011, 2017). Thus, in this study, the three epistemic levels describing the absolutist, multiplist, and evaluatist (Kuhn 1999; Kuhn and Park 2005) and the three epistemic dimensions describing limits of knowing, certainty of knowing, and criteria of knowing (Kitchener 1983) are interlaced to form a new framework for analyzing students’ epistemic views of model evaluation.

Furthermore, we examined the potential influence of the question contexts and the students’ grade levels. The same sets of questions were given to both eighth-grade and 11th-grade participants in order to understand the potential progression from eighth to 11th grade. It was hypothesized that students’ understanding of models and modeling would become more sophisticated with increasing age or increased learning in school. Past studies have shown that significant differences can be found between students of middle school and high school levels, while major progression was not likely to be found between students who are close in grade levels (e.g., between seventh and eighth grades). Among the studies conducted in different countries, consistent research findings have shown that students of 10th/11th grade had higher levels of views of models and modeling than seventh/eighth grade students (Grosslight et al. 1991; Krell et al. 2015; Lee 2018; Lee et al. 2017). However, whether this pattern can be found in terms of students’ understanding of model evaluation, and what qualitative differences can be found between age groups require further investigation.

Additionally, past research has suggested that epistemic views or epistemic criteria are domain-specific and context-sensitive (diSessa 2002; Krell et al. 2014; Lee and Tsai 2012). The different contextual information such as the characteristics of the presentation and the intended tasks could evoke different responses (Barzilai and Eilam 2018; Danish and Saleh 2015). In order to explore whether students’ understanding of model evaluation is context-specific, two question contexts, the Severe Acute Respiratory Syndrome (SARS) and dinosaur extinction were provided. In this study, models refer to mechanisms for explaining or predicting scientific phenomena (i.e., how diseases are transmitted or what caused the extinction of dinosaurs).

Students answered two sets of model evaluation questions based on two scientific contexts. After reading the information about the two models, the students had to choose from three epistemic assumptions and then provide written justifications explaining their choice of assumptions. Seeing students’ responses to model evaluation through the integrated view of epistemology, we posed the following research questions:

- RQ1a: What are the general trends of students’ choice of epistemic assumptions?
- RQ1b: How did the students justify their choice of epistemic assumptions?
- RQ2: To what extent do eighth-grade and 11th-grade students’ epistemic views of model evaluation differ?
- RQ3: To what extent were the students’ epistemic views of model evaluation consistent across different contexts?

### Views of and criteria for model evaluation

Schwarz et al. (2009), in conceptualizing the learning progression of modeling, identified two categories for “evaluating and revising models.” They suggested that *models need to be based on evidence about the phenomena*, and *models need to include only what is relevant to their purpose*. In their criteria, not only is evidence important for evaluating a model, so too is taking into account the modeling purpose (Schwarz et al. 2009; Schwarz and White 2005). Later, this framework was further expanded by Berland et al. (2016) who suggested an Epistemologies in Practice (EIP) framework. The knowledge construction and revision includes, for instance, explanation formation, argumentation, and modeling. One of the major aspects of EIP is “justification”—“How do we justify the ideas in our knowledge products?” At a higher epistemic level, it is expected that the students will construct, evaluate, and justify knowledge products by using available information such as data, scientific theories, and personal experiences.

Among the empirical studies on the categories and judgement criteria used by students for evaluating scientific models, most researchers used survey questionnaires or designed model evaluation tasks. Particularly, visual representations of models were highly emphasized. Al-Balushi (2011) studied the students’ evaluation of the credibility of scientific models and their corresponding textbook representations. The representations with different levels of abstractness included photographs, microscopic representations and symbols. The students were asked to evaluate the presentation of models based on four criteria, namely, certainty, imaginary, suspicious, and denial. They found that the students rated theoretical entities such as electron clouds or photons at a highly suspicious-denial combinational level. Overall, across grades nine to 11, the students showed a decrease in the certainty level and an increase in the imaginary level of their epistemological perceptions of scientific models.

In another study, the students were also given different presentations of models and were asked to judge their utility for scientific research and for supporting learning (Lee et al. 2017). They were presented with two models with different representations and were asked to choose from the following three options: (1) model A is better, (2) model B is better, and (3) both models are useful. The goal of the study was to investigate the potential relationship between representational characteristics and the perceived utility of models. The study found that the students preferred 3D models for scientific research. They thought schematic models were better or the same as textual models for research as well as for learning. The most used criteria for judgement included *cognitive perspective* (e.g., “the picture helps me understand better” or “I prefer reading the text”), *presentation* (“the picture represents the information clearly”), and *representational features* (e.g., “3D is more realistic”).

In Pluta et al. (2011) study, a more comprehensive list of students’ criteria for judging “good models” was provided. They designed a series of tasks for the students to compare different representations of models for the same phenomena. The study included two parts. In the first part, the participating seventh-grade students were first presented with different representations (e.g., flowchart, written explanation, causal diagram, and pictorial model) and discussed with peers the difference between “what is a model” and “what is not a model.” In the second part, they needed to compare models with different attributes (e.g., descriptive or explanatory, different degrees of complexity, different amounts of detail, etc.) and to decide on a better model in general or for a particular purpose of modeling. Major categories of epistemic criteria included g*oal of models, model constituents, communicative elements, evidential criteria,* and *epistemic elements*. Each major category includes sub-categories. They found that “pictorial form (in *communicative elements*),” in addition to “clarity (in *communicative elements*)” and the “explanatory function of the model (in *goals of models*)” were the criteria most often mentioned by the students. Later, Barzilai and Eilam (2018) followed a research procedure similar to that of Pluta et al. (2011). Nevertheless, the epistemic criteria used by the students were condensed into the three major categories of *communicative criteria, representational criteria,* and *epistemic affordance criteria.* The communicative criteria refer to the relation between the visual representation and the viewer (e.g., clarity, ease of use, detail, or simplicity, etc.). The representational criteria address the relationship between the representation and the reference (e.g., adequacy, credibility, or realism, etc.). Finally, the epistemic affordance criteria refer to whether the visual representation enables the viewers to achieve their epistemic goals, such as understanding, inquiry, or learning, etc. Barzilai and Eilam (2018) found that different designs and the inclusion of information in the scientific visual representations could evoke different evaluative criteria. However, only a minority of students were concerned about the validity of information and the source trustworthiness of the scientific representations.

In another group of studies, researchers studied how the students evaluated models during the process of modeling. The students’ criteria for judging model evaluation were interpreted through classroom observation or from the students’ worksheets. Schwarz et al. (2009) found that the students attended to features of their constructed models, including the level of abstraction of models, the audience and clarity of communication, and evidence, when constructing and revising models. Cheng and Brown (2015) provided the students with scaffolds regarding the criteria of visualization and exploratory power for evaluating models. They found that other self-generated criteria, such as more details, understandability, the nature of explanation, and consistency with other ideas were also adopted by the students.

In sum, different studies have generated overlapping criteria; however, the criteria were not entirely identical given the different research purposes of each study. Researchers have also found that scientists evaluated the quality of models based on criteria with a wider spectrum, such as “having high levels of conceptual coherence and clarity,” “compatible with theories in other fields,” “appropriately parsimonious,” “consistent with empirical evidence,” and “having a history of making novel empirical predictions” (cited from Pluta et al. 2011). Thus, to summarize, we have synthesized in Table 1 the criteria from some studies into four major categories: (1) representation and visualization, (2) scientific theory, (3) scientific evidence, and (4) purpose or epistemic aim of modeling.

**Table 1:** A summary of the criteria for model evaluation

Synthesized themes	Schwarz et al. (2009)	Al-Balushi (2011)	Pluta, Chinn and Duncan (2011)	Barzilai and Eilam (2018)	Criteria used by scientists (cited from Pluta et al. 2011)
Representation and visualization		Credibility of scientific models: certainty, imaginary, suspicious, and denial	Model constituents; communicative elements	Communicative criteria; representational criteria	Having high levels of conceptual coherence and clarity; appropriately parsimonious
Scientific theory					Compatible with theories in other fields
Scientific evidence	Models are based on evidence about the phenomena		Evidential criteria		Consistent with empirical evidence
Purpose or epistemic aim of modeling	Models need to include only what is relevant to their purpose		Goals of models	Epistemic aim affordance criteria	Having a history of making novel empirical predictions
Others			Epistemic elements		

### Personal epistemology and science learning

There are different conceptualizations and terminologies for personal epistemology. One commonly accepted categorization is to divide frameworks of personal epistemology into the unidimensional, developmental view of personal epistemology and the multi-dimensional view of personal epistemology (Deniz 2017; Feucht 2017). Seminal works on unidimensional frameworks include Perry’s (1970) nine-stage model of intellectual development, Baxter Magolda’s (1992) Espitemological Reflective Model, and King and Kitchener’s (2002) Reflective Judgement Model. In synthesizing and revising the previous categories of personal epistemic beliefs, Kuhn and colleagues (Kuhn 1993, 1999) suggested a developmental model for critical thinking that includes absolutist, multiplist, and evaluatist stages. Based on the work of Kuhn and colleagues (Kuhn 1999; Kuhn et al. 2000; Kuhn and Park 2005), from an absolutist view, knowledge is seen as an objective entity and as certain. It is located in the external world. Knowledge is the accumulation of a body of facts and it is knowable with certainty. Critical thinking is unnecessary because truth is readily discernable. From a multiplist perspective, knowledge is uncertain and subjective. It consists of opinions. Because everyone has the right to their own opinion, all opinions are equally right and critical thinking is irrelevant. Finally, the evaluatists view knowledge as uncertain but objective. Knowledge consists of judgments which require support in a framework of alternatives, evidence and argument. People can have different opinions, but opinions supported by evidence and argument have more merit than those that are not. Although Kuhn’s framework of epistemic understanding has been used in studying different aspects of science education, such as argumentation (e.g., Kuhn 1993) and scientific reasoning (e.g., Yang and Tsai 2010), the analysis of students’ understanding of models and modeling rarely adopts this framework.

One of the early developments of a multi-dimensional epistemic framework was the five dimensions of epistemic beliefs proposed by Schommer (1990), including the structure, certainty, source of knowledge, and the control and speed of knowledge acquisition. In later research, Hofer and Pintrich (1997) suggested that the dimensions of certainty of knowledge and simplicity of knowledge were under the area of nature of knowledge; and the dimensions of source of knowledge and justification of knowing were under the area of nature of knowing. These four-dimensional epistemic beliefs were widely applied in science education studies and have been used to investigate the relationships between epistemic beliefs and other factors, such as motivation, strategies for learning, and scientific inquiry skills (Ding 2014; Lee et al. 2016; Lising and Elby 2005).

Epistemic cognition is another multi-dimensional model of personal epistemology and is defined as “explicit or tacit cognitions related to epistemic or epistemological matters” (Chinn et al. 2011, p. 141). It also refers to thinking about knowing (Greene et al. 2010). Kitchener (1983) termed *epistemic cognition* in her three-level model of cognitive processing for solving ill-structured problems as interpreting the nature of an ill-structured problem and defining the limits of any strategies for solving it. In ill-structured problems, evidence, expert opinion, reason, and argumentation can be brought to bear on the issues, but no procedure can guarantee a correct or absolute solution. The three levels consist of the cognitive, the meta-cognitive and the epistemic cognition levels. Epistemic cognition, which is above the cognitive and the meta-cognitive levels, includes one’s reflections upon the *limits of knowing, the certainty of knowing,* and the *criteria of knowing*. Chinn et al. (2011) further suggested five components of epistemic cognition including epistemic aims and epistemic value, the structure of knowledge, the sources and justification of knowledge, epistemic virtues and vices, and reliable and unreliable processes for achieving epistemic aims. When facing conflicting information, the spontaneous epistemic cognition that people may be engaging in include assessing the validity of claims, considering justifications of claims, noting consistency between data and claims, and so on (Barzilai and Zohar 2016).

While the developmental models only represent one dimension of epistemology, and the multi-dimensional models do not suggest clear developmental stages, one possible solution is to combine both models and create a matrix view of personal epistemology. Feucht (2011 and 2017) developed an Educational Model of Personal Epistemology by integrating the two frameworks—Kuhn’s (1999) three developmental stages and Hofer’s (2000) four-dimensional model of epistemic beliefs (i.e., source, development, certainty, and justification). The matrix view suggests 12 cells incorporating different levels of development for different dimensions of personal epistemology. This matrix has been used to assess and identify teachers’ epistemic patterns in science teaching (Feucht 2011; 2017).

In the current study, we adopted an integrated framework similar to Feucht’s work (2017). Kitchener’s (1983) epistemic cognition dimensions rather than Hofer’s epistemic belief framework were combined with Kuhn’s (1999) framework. This research decision was based on the following reasons. First, science education researchers have argued that epistemic cognition plays an important role in scientists’ evaluation of the validity and accuracy of scientific products such as models and arguments (Kelly 2016; Longino 2002). Because the process of solving an ill-structured problem involves making judgements about arguments and evidence, evaluating information from inconsistent and imperfect data sources, and developing and arguing for a reasonable solution, we draw some similarities between the process of solving ill-structured problems and making judgements among multiple competing models. Second, although Kitchener’s (1983) model is not developmental, she emphasized that epistemic cognition could take different forms based on the underlying epistemic assumptions that are developed in the adolescent years. The different epistemic assumptions described by Kitchener (1983) resemble Kuhn’s three positions representing the objective/subjective epistemic views. Kitchener’s epistemic cognition can be seen as an early model that integrates the developmental and the multi-dimensional framework. We summarize in Table 2 the integrated framework based on our interpretation of the literature (Kuhn et al. 2000; Kuhn and Park 2005).

**Table 2:** An integrated framework for model evaluation combining Kuhn’s (1999) three developmental stages and Kitchener’s (1983) epistemic cognition dimensions

Dimensions of epistemic cognition	Developmental levels		
	Absolutist	Multiplist	Evaluatist
Limits of knowing	Reality is directly knowable; model is reality	Reality is not directly knowable; model represents facets or part of the reality	Reality is not directly knowable; model represents facets or part of the reality
Certainty of knowing	Models come from an external authority and are certain	Models are constructed internally and are uncertain	Models are constructed internally and are uncertain
Criteria for knowing	Comparing models to reality and determining whether they are true or false	Evaluating and comparing models based on opinions; critical thinking is irrelevant	Evaluating and comparing models based on criteria of argument and evidence

## Methods

### Model evaluation items

The model evaluation items in this study have the following three main characteristics: (1) they are contextualized by real scientific problems, (2) they present at least two competing models, and (3) they present three epistemic positions for the students to choose from. The design of the items in this study was inspired by previous item designs in the literature. The items were to find out students’ views regarding the questions: “Is there a way to decide whether one model is better than another?” and “What are the criteria for the evaluation of a model?” (Schwarz and White 2005; Sins et al. 2009). In an earlier developed item for model evaluation, students were asked whether they agreed or disagreed with the following statement: “Since scientists disagree about why dinosaurs became extinct, it’s clear that no one understands exactly how it happened. Therefore, any scientific model or theory of how it happened is just as good as any other” (Schwarz and White 2005, p. 190). In this study, we focused on similar logic of inquiry but provided the students with a description of competing models in ill-defined contexts. This design is consistent with a recent trend of assessing students’ understanding of models in which different representations are shown to students for comparison and for evoking deeper thinking (Al-Balushi 2011; Torres and Vasconcelos 2015; Lee et al. 2016).

Two models of infection for the Severe Acute Respiratory Syndrome (SARS) virus were presented in the first question set, and two explanatory models of dinosaur extinction were presented in the second question set (see "Appendix" section). We intentionally selected these two non-textbook science contexts to avoid students answering based on the *right answers* taught in school. These two science questions were, nevertheless, still comprehensible at the middle school level. Because scientists are still attempting to understand more about the mechanisms or causes of these two events, both questions possessed high levels of uncertainty. While many competing explanatory models are available for both cases, they are used as stimuli to probe the students’ epistemic thinking model evaluation.

After reading the information about the two models, the students had to choose from three epistemic assumptions: (1) “one model is better than another”; (2) “both models can be valuable; there is no need to decide which model is better” (herein after referred to as “both models can be valuable”); and (3) “we cannot know which model is better unless new evidence supports one of them” (hereinafter referred to as “depends on the evidence). The three assumptions correspond to the *absolutist*, *multiplist*, and *evaluatist* views, respectively. The three assumptions were written based on Kuhn’s three stages of epistemic levels and were revised from Schwarz and White’s (2005) categories of students’ responses to model evaluation. The students also needed to provide written justifications explaining their choice of epistemic views.

The two sets of questions were reviewed by and discussed among the co-authors of this study. An additional two middle school teachers and one high school teacher were also invited to review the items. The review process was to further ensure the construct validity, face validity, and content validity of the items.

### Data collection and data analyses

In this study, we surveyed 101 eighth graders (including 60 females) and 87 11th graders (including 42 females) from the central and southern parts of Taiwan. The entire questionnaire was completed online in computer labs.

#### Quantitative analyses

The students’ choices among the three epistemic assumptions were tallied. Then the percentages were calculated by grade and by the epistemic view chosen for model evaluation. Comparisons were further made between grades and across epistemic views. Because all items were categorical, we conducted Chi-square independent tests for understanding the differences within the same educational level or between educational levels. We also used McNemar-Bowker tests (Elliott and Woodward 2006) to examine the consistency of the students’ answers across different contexts.

#### Qualitative analyses

The qualitative analyses consisted of four major steps, open coding, theme developing and mapping, final coding, and data triangulation. First, open-coding methods were applied to students’ written responses, and a list of free codes was created. This process was to gain an understanding of the data and apply labels to the data. Second, the free codes were condensed and mapped into the integrated framework of Table 2 to form coding themes. In other words, each coding theme was identified with the epistemic levels as well as the epistemic dimensions. The draft of coding themes was then tested by two coders on the data and modified until the coding themes were saturated. Any discrepancies of coding were discussed among the two coders until consensus was reached. A list of coding categories and examples is given in Table 3, and the corresponding dimensions and levels are also marked. During the process of testing the themes and final coding of the data, two coders independently coded 10–20% of the data three times. The inter-rater reliability of the items was between 0.81 and 0.84.

**Table 3:** A list of coding themes for an integrated view of model evaluation

Coding themes	Definition	Example	Dimension	Developmental level
Value (negative)	Refers to the criticism of lack of value when inquiring into knowledge	Dinosaurs’ extinction happened a long time ago. Even if we can know more about it, the knowledge won’t be useful It is meaningless to make more hypotheses or models about dinosaur extinction. We won’t change anything	LK	M, E
Method (limit)	Refers to the perceived limitation of science methods when inquiring into knowledge	It is not likely that we will find new evidence We did not live in ancient times, so it is not possible to know about it [dinosaur extinction]	LK	M, E
Certain	Refers to the belief of certainty of science. There is only one right explanation of science phenomena	It’s better not to change often. It is chaotic There’s only one truth	CerK	A
Uncertain	Refers to the belief of the temporary and multiplicitous nature of science. There might be multiple explanations for the same phenomenon	There are multiple explanations	CerK	M, E
Authority	Refers to the belief that knowledge comes from people with authority, such as scientists or science teachers	They [models] are all true because they are all stated by scientists	CriK	A
True/false	Refers to the personal judgement of whether the statement is correct or incorrect	A volcanic explosion is the most likely cause of dinosaur extinction	CriK	A
Pragmatic	Refers to the perceived purposes and usefulness of models such as representation and communication, explanation, prediction, and problem solving	Model A is better because it covers more details As long as a model can help us to understand the disease better and help to produce antigens, it is a good model. No need to decide which one is better	CriK	M
Evidential	Refers to using evidence to support arguments and its inquiry process	It depends on whether new evidence is found As the technology evolves, there is new evidence available	CriK	E
		I don’t know. Based on my intuition		
Not applicable (NA)			NA	

*LK* limit of knowing, *CerK* certain of knowing, *CriK* criteria of knowing, *A* absolutist, *M* multiplist, *E* evaluatist

**Fig. 1:**
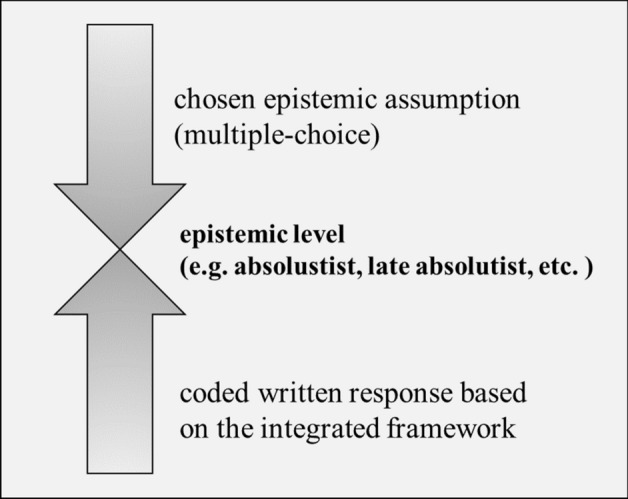
An illustration of the final coding process. Comparisons were made between each student’s choice of epistemic assumptions and his/her coded written response in order to confirm their epistemic level

Finally, after completing the coding, two coders adopted a middle-out approach to confirm students’ epistemic levels (see Fig. 1). Through triangulating both sources of data, we compared each student’s choice of epistemic assumptions (i.e., the three options from the multiple-choice questions)and his/her coded written response in order to confirm their epistemic level. When the students’ written responses were more or less sophisticated than the choice of the three developmental assumptions, a new epistemic level emerged. For instance, one student chose “one model is better than the other” (an absolutist assumption) and stated that it is important that the better model is supported by scientific evidence. In acknowledging both the choice of epistemic assumption and the written explanation to the assumption, we believed that this student had already developed thinking beyond an absolutist view, and thus assigned his answer to the late absolutist category. This approach allowed us to consider both the perspectives from the forced-choice response and the participating students’ free responses, thus providing insights that may have been overlooked in past studies. In this study, four new adjusted developmental positions, *late absolutist, early multiplist, late multiplist,* and *early evaluatist* emerged from the data. Details are described in the "Results" section.

#### Coding themes

As shown in Table 3, the LK category includes the *value* and *method* sub-categories. These two sub-categories reflect the students’ concerns about lack of value and limitation of scientific methods when inquiring into knowledge. The CerK category includes *certain*, *uncertain,* and *authority.* The *certain* sub-category emphasizes that the model is certain and only one right model exists. The *uncertain* sub-category focuses on the belief that models are tentative and that multiple models exist. The *authority* sub-category refers to the belief that knowledge is from an external authority, such as scientists. The CriK category also includes three sub-categories, namely, t*rue/false, pragmatic,* and *evidential. True/false* refers to the personal judgement of the correctness of the description of the model; *pragmatic* refers to the usefulness and purposes of models; and *evidential* refers to using scientific evidence to support the evaluation of models.

## Results

First, we present the distribution of the three epistemic assumptions based on the forced-choice question (RQ1a). Then, we present the results of coding of the written responses based on the integrated view of the personal epistemological framework and their final coded epistemic levels (RQ1b). The differences in grade levels (RQ2) and the comparisons of the two contexts (RQ3) are presented together in this section. Finally, to further clarify, we highlight and summarize the results.

### Overall trend of students’ chosen epistemic assumptions (multiple-choice questions)

Table 4 shows that nearly one fifth of the eighth and 11th grade students believed that *“one model is better than another*”. In other words, the majority of the students chose a more sophisticated view. However, the two contexts seemed to lead to different choices of the *“depends on the evidence”* and *“both models can be valuable”* assumptions. In the SARS case, the majority of 11th and eighth grade students, nearly 70% of the eighth grade students and 57.47% of the 11th graders, thought that both explanations can be valuable. Nevertheless, while the eighth-grade students were still more likely to choose a *“both models can be valuable”* than *“depends on the evidence”* for the dinosaur extinction question, the most chosen answer for the 11th-grade students was an evaluatist assumption (45.98%). However, within the same context, the results of chi-square analysis showed no statistical significant relationships between students’ educational levels and their epistemic views of model evaluation.

**Table 4:** The percentage of students choosing the three epistemic assumptions and results of chi-square tests

Biological concept	Eighth grade %			Eleventh grade %			Education level χ^2^
	One is better than another	Both models can be valuable	Depends on the evidence	One is better than another	Both models can be valuable	Depends on the evidence	
SARS	15.53	69.90	14.56	18.39	57.47	24.14	3.65
Dinosaur extinction	20.79	44.55	34.65	19.54	34.48	45.98	2.73

*p < 0.05, **p < 0.01, ***p < 0.001

Further analysis with McNemar-Bowker tests also confirmed that the context of the item influenced students’ epistemic views in model evaluation (χ^2^ = 23.75, *p* < 0.001 for eighth-grade students; χ^2^ = 13.43, *p* = 0.004 for 11th-grade students). Only 50.4% of the eighth-grade students chose the same answers for the two questions; an even lower percentage (40.2%) of the 11th-grade students had consistent answers for the two contexts. A high percentage of students who chose a *“both models can be valuable”* assumption for the SARS question shifted their views to *“depends on the evidence”* when it came to the dinosaur extinction question.

### Students’ epistemic views of model evaluation based on the integrated framework

About 55–60% of eighth-grade students (60.3% for the SARS context and 55.4% for the dinosaur context) and about 75% to 80% of the 11th-grade students (80.3% for the SARS context and 75% for the dinosaur context) provided meaningful written responses justifying their choice of developmental assumptions. More 11th-grade than eighth-grade students were able to provide meaningful justification. The rest of the students left the written part of the question blank or provided answers that were irrelevant to their view of model evaluation (e.g., “I don’t know” or “Because I thought so”). In the following, we illustrate the students’ views of model evaluation based on both of their forced-choice responses and their written justifications. The results were presented by the three major groups of epistemic levels.

#### Absolutist and late absolustist

**Fig. 2:**
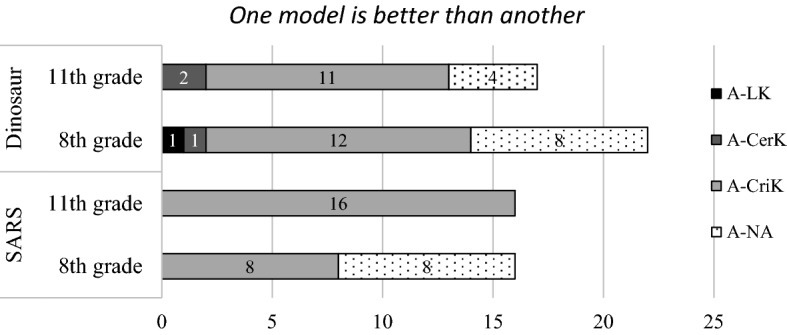
The students’ thinking in the LK, CerK, and CriK categories in relation to the absolutist and late absolutist levels

As shown in Fig. 2, the students who chose “one model is better than another” mainly focused on CriK, while few students reflected upon LK and CerK. The overall trends were similar across the two question contexts and were also similar for the eighth- and 11th-grade students. In the CriK dimension, the majority of the students (N = 35) commented on whether the content in the two explanatory models is true or false (coded as *true/false*; see Table 3). For instance, one eighth-grade student commented that “If SARS was air-borne, then everyone should be infected by now. So it cannot be right.” An 11th-grade student stated that “I think climate change sounds like the cause [for dinosaur extinction]”. During data coding, we did not take into account whether students’ judgments were scientifically correct or not.

In addition to absolutist, we shaded 13 students in Table 5 and renamed those students as *late absolutist* which the typical epistemic views in Table 2 do not account for*.* Although these students chose “one mode is better than the other,” they may hold a more sophisticated epistemic view than the students who were categorized as absolutist. We found the *late absolutist* justified the absolutist view by more sophisticated thinking regarding LK (i.e., limits of methods), CerK (i.e., uncertain) and CriK (i.e., pragmatic and evidence). For instance, one student who considered by the perceived limitation of current science methods for the Dinosaur model (coded as method(limit)* in LK). In another example, one student stated that “there are multiple causes for dinosaur extinction” (coded as *uncertain*).

**Table 5:** Students’ epistemic views for model evaluation—absolutist and late absolutist levels

Dimensions	Coding themes	SARS		Dinosaur	
		8th	11th	8th	11th
Limits of knowing	Method (limit)^b^			1	
Certainty of knowing	Certain			0	2
	Uncertain^a^			1	
Criteria for knowing	True/false	3	15	7	10
	Pragmatic^a^	4	1	3	
	Evidential^a^			2	1
NA		8		8	4
Total		16	16	22	17

Late absolutist level marked with superscripts. The rest of the students in this table are at absolutist level

^a^Late absolutist

^b^Context specific

A few students utilized criteria for knowing that is considered for more sophisticated levels. Seven eighth-grade students and one 11th grader based their evaluation of the models on how well they served the purposes of models (coded as *pragmatics*). For example, one student chose one SARS model as being better than the other because “one model represents more clearly than the other.” Additionally, four were concerned about the quality of the evidence or emphasized the method of inquiry (coded as *evidential*). For example, one student stated that “All hypothesized models [in the dinosaur context] are possible, but one of them must have more evidence than the others.” Another student argued that one should judge that “one model is better than the other through engaging in archaeological investigation and scientific reasoning.”

#### Early multiplist, multiplist, and late multiplist

**Fig. 3:**
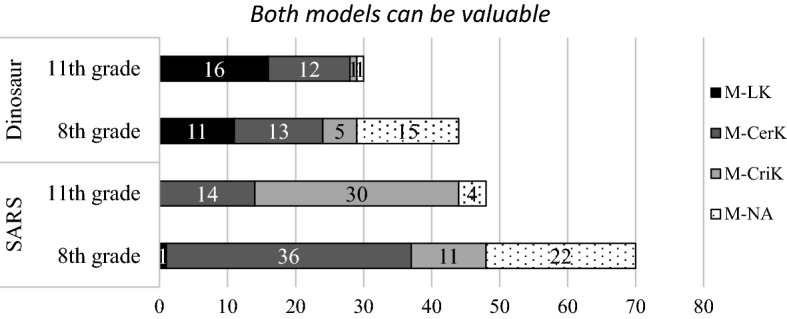
The students’ justifications of multiplist views presented by the LK, CerK, and CriK categories

The students who chose “both models can be valuable” approached the question in the dinosaur context differently from how they approached it in the SARS context. When answering the dinosaur questions, the majority of students’ justifications were based on LK and CerK, and only a small number were concerned with CriK (see Fig. 3). However, the majority of the students thought of CerK and CriK when they answered the model evaluation questions in the SARS context.

The students’ evaluation of the dinosaurs models were closely related to their perceptions of dinosaurs; therefore, we marked them as context-specific (marked with an asterisk) in Table 6. The context-specific justifications were shown in two types of responses. First, some students, both in eighth and 11th grade, questioned the value of pursuing the answer of what caused dinosaurs to become extinct (coded as *value *(*negative*)*; n = 6). These students argued that because dinosaurs became extinct a long time ago, there is no need to find the answer. For instance, one 11th grader wrote “It all happened a long time ago. Even if we can find the answer, it won’t be valuable.” Second, a large number of the students chose “no need to decide which model is better” because they thought no evidence can be found. We coded this passive attitude as *method *(*limit*)* in order to differentiate it from the more positive attitude of finding evidence.

**Table 6:** Students’ epistemic views for model evaluation—early multiplist, multiplist, and late multiplist levels

Dimensions	Coding themes	SARS		Dinosaur	
		8th	11th	8th	11th
Limits of knowing	Value (negative)^c^				6
	Method (limit)^c^			11	10
	Method (limit)	1			
Certainty of knowing	Uncertain	36	14	13	12
Criteria for knowing	True/false^a^	6	8	2	
	Authority^a^		1		
	Pragmatic	5	18		
	Evidential^b^		3	3	1
NA		22	4	15	1
Total		70	48	44	30

Early multiplist and late multiplist levels are marked with superscripts. The rest of the students in this table are at multiplist level

^a^Early multiplist

^b^Late multiplist

^c^Context specific

When the students answered the SARS questions, only one student mentioned LK and the others’ justifications focused on CerK and CriK. Despite the large number of students believing in uncertainty, more students justified their views of model evaluation by CriK in the SARS context than in the dinosaur context. Students’ responses regarding the uncertainty (coded as *uncertain* in CerK) included, for example, “there could be more than one explanation” and “each model has its advantages.” Some students, especially the 11th graders, emphasized the explanatory and predictive purpose of models (coded as *pragmatic*). For instance, one 11th-grade student stated that “because both models can be used to predict how a virus infects people, both can be right.” Similarly, an eighth-grade student stated that “both [models] clearly explain the pathway of how SARS is transmitted.” Because the pragmatic criteria can be seen as personal opinions supporting one’s judgement, it is consistent with the *mulitplist* level.

The trends in the context of SARS also differed for the eighth- and 11th-grade students. In the SARS context, eighth-grade students were more likely to think of the uncertain nature of science than the judging criteria, while the 11th-grade students were more likely to be concerned with the criteria of knowing (CriK) than with uncertainty (CerK). Additionally, 11th-grade students were more able than eighth graders to provide meaningful justifications in both contexts. Although a great number of middle school student responses were coded as the uncertain nature of science, the answers were simple (e.g., “both can be right”).

Two additional epistemic levels, *early multiplist* and *late multiplist* emerged from the data. The justifications based on authority (coded as *authority* in CerK) and the personal judgments of true or false (coded as *true/false*) are close to absolutist thinking; thus, we further marked them in shades in Table 5 and labeled them as *early multiplist*. For example, one 11th-grade student stated that “[both models can be true] because “they are both true because they are both stated by scientists.” Nevertheless, seven students stated that all models need to be supported by evidence. Hence, they are categorized as *late multiplist*.

#### Early evaluatist and evaluatist

**Fig. 4:**
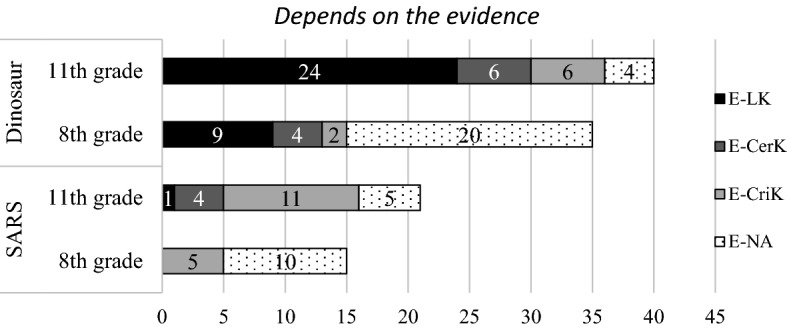
The students’ justifications of evaluatist views presented by the LK, CerK, and CriK categories

The students’ justifications for the epistemic assumption of “depends on the evidence”distributed in LK, CerK, and CriK mainly focused on the limitation of science methods [coded as *method *(*limit*)*], true/false of the science content (coded as *true/false*), the uncertain nature of science (coded as *uncertain*), and the scientific evidence (coded as *evidence*). The patterns of the students’ justification were different in the dinosaurs and the SARS contexts (as shown in Fig. 4 and Table 7). On the one hand, in the SARS context, 10 students (eight at 11th grade and two at eighth grade) emphasized the importance of using evidence to decide which model is better. For example, one 11th-grade student justified his choice as follows: “Now both models are hypothesized by scientists. Therefore, evidence is needed to decide which one is better.” On the other hand, in the dinosaur context, two 11th-grade students also stated that new evidence was needed (coded as *inquiry*) but 33 students (24 at 11th grade and nine at eighth grade) were concerned with the lack of evidence [coded as *method *(*limit*)*]. For instance, one student stated, “We don’t have direct evidence yet to find out the real reasons why dinosaurs became extinct.” These concerns of the limitation of scientific methods were specific to the dinosaur context as well.

**Table 7:** Students’ epistemic views for model evaluation—early evaluatist and evaluatist levels

Dimensions	Coding themes	SARS		Dinosaur	
		8th	11th	8th	11th
Limits of knowing	Method (limit)^b^			9	24
	Method (limit)		1		
Certainty of knowing	Uncertain		4	4	6
Criteria of knowing	True/false^a^	3	3	2	4
	Evidential	2	8		2
NA		10	5	20	4
Total		15	23	35	40

Early evaluatist is marked with superscripts. The rest of the students in this table are at evaluatist level

^a^Early evaluatist

^b^Context specific

In both contexts, a few students mentioned that multiple models are possible (coded as *uncertain*) and knowledge is uncertain. This view of model evaluation is still consistent with the evaluatist view. However, a few students who agreed with “we cannot know which model is better unless new evidence supports one of them” justified this epistemic assumption by the simple right/wrong one of the two models (coded as *true/false*) in the written response but did not consider the purposes of model or the need of scientific evidence. Thus, we considered these students as *early evaluatist* and considered that they may not yet have a mature evaluatist understanding. Finally, compared to the students who chose the previous two views, a higher percentage of students in the eighth grade who chose the evaluatist views could not provide meaningful justifications. These students also might not have gained full understanding of model evaluation from an epistemic perspective.

## Conclusions

We conclude and highlight the major findings of this study as follows:

### Differences in grade levels

- In both contexts there are higher percentages of 11th-grade students choosing the evaluatist assumptions than eighth-grade students; however, there is no statistically significant difference between the two grades
- A higher percentage of 11th-grade than eighth-grade students could provide meaningful justifications for their choice of epistemic assumptions
- For students choosing “both models are valuable” and “depends on the evidence” assumptions, the 11th-grade students were more likely than the eighth-grade students to think in terms of pragmatic and evidential criteria.

### Effects of the context

- Results showed that the students’ choices of the three epistemic assumptions were statistically significantly different in the two contexts. About 50% of the students had different epistemic views in the two contexts
- The written responses provided by the students showed that the context of the question can evoke certain responses. The dinosaurs context evoked the students’ concerns of the value of knowledge and the limit of scientific methods in futher inquiring into the models of dinosaur extinction.

### Consistency between chosen epistemic assumptions and the justifications

- Although the *evaluatist* position is considered as a more sophisticated epistemic view, some of the students provided justifications that were inconsistent with an evaluatist view. Thus, we categorized those students as having an *early evaluatist* view of model evaluation. Nevertheless, the students using evidence to justify absolutist or multiplist asumptions were catgorized in this study as having *late absolutist* or *late multiplist* views
- While some students, particularly in 11th grade, who had *multiplist* views of model evaluation tended to justify their assumptions by the usefulness and purposes of the models (coded as *pragmatics*), other students justified their assumptions based on true/false judgement or opinions from authorities (identified as *early multiplist*).

## Discussion

Results showed a general tendency of students choosing “one model is better than another” justifying them by true/false and pragmatic criteria of knowing; students who chose “both models are valuable” tended to justify them by the certainty of knowing and the pragmatic criteria; and the students who chose “depends on the evidence” tended to think of the evidential criteria of knowing and the limits of knowing. However, the results also showed that students changed their views of models when different contexts were provided. A high percentage of students who answered “both models can be valuable” for the SARS question shifted their views to “depends on the evidence” when it came to the dinosaur extinction question. This finding confirmed that students’ development of personal epistemology can be dynamic as it is influenced by factors such as the context, affect, or cognitive ability (Bendixen and Rule 2004). Another interesting observation was that some students who held *multiplist* and *evaluatist* views questioned the value of evaluating the dinosaur extinction model and also had doubts about the plausibility of finding scientific evidence. Researchers have found that some knowledge seems to be more valuable or significant than other knowledge due to many different reasons, such as being incomplete, being personally irrelevant, or being useful for solving societal problems (Chinn et al. 2011). In this study, we found that some students believed that knowing why dinosaurs became extinct was meaningless and impossible. Thus, this belief may have guided the evaluators to adopt criteria for model evaluation that may be different from those used in other more meaningful science contexts. This might explain why we found inconsistent choices of epistemic views in the two contexts.

Our results also showed that the overall students’ epistemic views for model evaluation were somehow consistent with research findings of the general development of students’ epistemic beliefs. Past studies have found that students’ understanding of models and modeling in general progressed from middle school to high school (e.g., Lee 2018). In the current study we found that 11th-grade students were more likely to provide meaningful justifications and were more likely to use pragmatic and evidential criteria of knowing than were eighth-grade students. We also found that in both grades, the majority of the students held a *multiplist* level of modeling (including early multiplist and late multiplist), which is consistent with past research. In separate studies, Yang and colleagues (Yang 2005; Yang and Tsai 2010) found that at eighth and 11th grade, most students tended to hold multiplist beliefs of science. Thus our result indicates that without particular instruction of modeling, the students’ judgement of model evaluation was perhaps largely influenced by their general scientific epistemic beliefs. This finding sheds some light on the mutual relationships between modeling-based epistemic beliefs and science-general epistemic beliefs.

In this study, we have suggested a new system to understand what underlies students’ evaluation of models from personal epistemology. In the three dimensions of the integrated framework, we found that relatively fewer students referred to the *limits of knowing,* and even when they did, they referred to the lack of value or the limit of methods for investigating dinosaurs. Students’ epistemic awareness of the limitations of models is an important aspect of the understanding of models and modeling. In the Next Generation Science Standards (National Research Council 2012), it is stated that “Because all models contain approximations and assumptions that limit the range of validity of their application and the precision of their predictive power, it is important to recognize their limitations” (p. 52). This is not only an aspect that has not been emphasized in previous studies, but it also has important implications for teaching modeling. This result reflects that in science classes students probably do not have opportunities to explore and reflect upon the limitations of models. Future modeling-based instruction should not only help students to construct and revise models, but should also facilitate the students’ evaluation of the limitations of models.

In the *criteria of knowing* dimension, some students, 11th graders in particular, mentioned the pragmatic criteria. On the one hand, we found that the pragmatic criteria coincide with the criteria in the previous studies, such as the “goals of models” criteria in Pluta et al.'s (2011) framework, as well as Barzilai and Eilam's (2018) representational and epistemic aim criteria. In a way, this integrated framework can be seen as an expansion of the previous criteria, while the previously developed criteria can be subsumed under the CriK dimension. On the other hand, the results can be an indication that these students had begun to develop some understanding of the purposes and aims of scientific models, which is essential for the competence of model evaluation (Schwarz et al. 2009).

The emergence of additional epistemic levels based on the integrated analytical framework not only provides more *in between* categories, but also suggests that the progression of students’ epistemic views of models might not be linear. For example, the students who believed that one model is better than the other also believed in using scientific evidence to prove which one is the best. In other words, before the students make the transition to truly evaluatist, the absolutist view and the evaluatist view can co-exist. This is a developmental perspective that is different from the traditional developmental view of epistemology. Taking King and Kitchener’s (2002) Reflective Judgement Model for example; the seven developmental stages are summarized into three levels (i.e., pre-reflective thinking, quasi-reflective thinking, and reflective thinking). However, they argued for a natural logic to the progression whereby one stage builds on the previous stage. One cannot reach stage 3 until being fully aware of stage 2. Whether our conceptualization of students’ epistemic development is more productive than the traditional frameworks awaits further investigation and application in future studies.

The current study has some limitations. First, we found that using both forced-choice questions and open-ended responses can better discover the complex nature of students’ epistemic views than using forced-choice questions alone. However, because some students were not used to open-ended questions and because of the philosophical nature of the questions, some, particularly those in eighth grade, did not provide meaningful written responses. Future studies can consider other qualitative research methods, such as using in-depth interviews or classroom discourse to elicit students’ thinking. Second, because of the exploratory nature of the study, our integrated framework was only tested with a limited number of students. We expect further modification or expansion of the framework when it is applied to larger samples or tested on different ill-defined science dilemmas for model evaluation.

## Appendix

### Model evaluation items

1. The Severe Acute Respiratory Syndrome (SARS) infected many people a few years ago and caused a high mortality rate. Scientists had two different models to explain the route of infection:
  Model 1: people are infected by the SARS virus which is spread by a SARS patient. The virus is spread through coughing or sneezing at a close distance
  Model 2: people are infected by inhaling the SARS virus that is already in the air
  - Given the two models suggested by the scientists, which of the following statements do you most agree with?
    1. One model is better than the other
    2. Both explanations can be valuable; there is no need to decide which model is better
    3. We cannot know which model is better unless new evidence supports one of them
  - Please explain why you agree with the statement

2. Scientists have developed different models for explaining the extinction of dinosaurs. One of the models focuses on the existence of alkaloid-rich flowering plants about 120 million years ago. Those plants were poisonous to the dinosaurs. The dinosaurs developed physiological disorders due to eating the poisonous plants and eventually became extinct. In another model, the scientists focused on the impact caused by an asteroid about 65 million years ago. The ash generated by the impact filled the sky and caused a drastic drop in the temperature on earth which lasted for years. The extreme weather and lack of sunlight changed the environment which led to the death of the dinosaurs.
  - Given the two models suggested by the scientists, which of the following statements do you most agree with?
    1. One model is better than the other
    2. Both explanations can be valuable; there is no need to decide which model is better
    3. We cannot know which model is better unless new evidence supports one of them
  - Please explain why you agree with the statement.
